# Dementia subtype and living well: results from the Improving the experience of Dementia and Enhancing Active Life (IDEAL) study

**DOI:** 10.1186/s12916-018-1135-2

**Published:** 2018-09-11

**Authors:** Yu-Tzu Wu, Linda Clare, John V. Hindle, Sharon M. Nelis, Anthony Martyr, Fiona E. Matthews, on behalf of the Improving the experience of Dementia and Enhancing Active Life study, L. Clare, I. R. Jones, C. Victor, J. V. Hindle, R. W. Jones, M. Knapp, M. Kopelman, R. Litherland, A. Martyr, F. E. Matthews, R. G. Morris, S. M. Nelis, J. Pickett, C. Quinn, J. Rusted, J. Thom

**Affiliations:** 1 REACH: The Centre for Research in Ageing and Cognitive Health, St Luke’s Campus, University of Exeter Medical School, Exeter, EX1 2LU UK; 2 Institute of Health and Society, Newcastle University, The Baddiley-Clark Building, Richardson Road, Newcastle Upon Tyne, NE4 5PL UK; 3 Present address: Health Service and Population Research Department, Institute of Psychiatry, Psychology and Neuroscience, King’s College London, David Goldberg Centre, De Crespigny Park, Denmark Hill, London, SE5 8AF UK

**Keywords:** Dementia, Subtype, Quality of life, Wellbeing, Life satisfaction, Caregiving

## Abstract

**Background:**

The heterogeneity of symptoms across dementia subtypes has important implications for clinical practice and dementia research. Variation in subtypes and associated symptoms may influence the capability to live well for people with dementia and carers. The aim of this study is to investigate the potential impact of dementia subtypes on the capability to live well for both people with dementia and their carers.

**Methods:**

The analysis was based on the 1283 dyads of community-dwelling people with dementia and carers in the Improving the experience of Dementia and Enhancing Active Life (IDEAL) project, a large cohort study in Great Britain. Capability to live well was defined using three measures: quality of life, life satisfaction and wellbeing. Structural equation modelling was used to investigate capability to live well in seven dementia subtypes: Alzheimer’s disease (AD), Vascular dementia (VaD), mixed AD/VaD, frontotemporal dementia (FTD), Parkinson’s disease dementia (PDD), Lewy body dementia (LBD) and unspecified/other, accounting for dyadic data structure and adjusting for age and sex, type of relationship between person with dementia and their carer and the number of chronic conditions.

**Results:**

The major subtypes in this study population were AD (56%), VaD (11%) and mixed AD/VaD (21%). Compared to participants with AD, people with non-AD subtypes generally reported a lower capability to live well. Carers for people with PDD (− 1.71; 95% confidence interval (CI) – 3.24, − 0.18) and LBD (− 2.29; 95% CI – 3.84, − 0.75) also reported a lower capability to live well than carers for people with AD. After adjusting for demographic factors and comorbidity, PDD (− 4.28; 95% CI – 5.65, − 2.91) and LBD (− 3.76; 95% CI – 5.14, − 2.39) continued to have the strongest impact on both people with dementia and their carers.

**Conclusions:**

This study suggests a variation in capability to live well across dementia subtypes. It is important for care providers to consider different needs across subtypes. Health professionals who provide post-diagnostic support may need to pay more attention to the complex needs of people living with PDD and LBD and their carers.

**Electronic supplementary material:**

The online version of this article (10.1186/s12916-018-1135-2) contains supplementary material, which is available to authorized users.

## Background

Dementia is a key priority area in health and social care planning across the world, with an increasing emphasis on timely diagnosis and appropriate support throughout the trajectory of the condition [1, 2]. To support the large number of people living with dementia and their carers to live well and manage the condition, provision of effective post-diagnostic care has become an important issue for health services and clinical practice [3]. For example, in the UK the current National Health Service (NHS) strategy to improve dementia care includes encouraging general practitioners to play a leading role in the coordination and continuity of care for people with dementia [4]. Yet there is a lack of evidence-based guidance to enable health professionals to identify high-risk groups who might experience poor quality of life due to dementia-related symptoms and need additional support to live well with their condition.

Living well with chronic illness has been defined as the best achievable state of physical, mental and social health and wellbeing, indexed by a self-perceived level of comfort, function and contentment with life [5]. The concept of ‘living well’ with dementia has been largely equated to a good quality of life. However, living well can mean more than a score on quality of life measured at a specific time point. The concept should encompass other inter-related constructs such as the experience of satisfaction with life and a sense of subjective wellbeing [6].

The heterogeneity of symptoms across dementia subtypes has been a key topic in clinical practice and relevant research [7, 8]. Variation in the symptoms associated with different subtypes may influence quality of life and wellbeing in people with dementia and their carers. Needs for post-diagnostic support and care may also vary across different subtypes. Indeed, previous studies have highlighted the potential impact of dementia subtypes on quality of life in people with dementia and on the burden of caregiving. Compared to Alzheimer’s disease (AD), people with Lewy body dementia (LBD) tend to report a worse quality of life [9–11]. Family carers for people with frontotemporal dementia (FTD) and LBD have been found to experience a greater caregiving burden than carers for people with AD [11–13]. However, most existing studies were based on a relatively small number of participants recruited from clinical or residential settings and focused on specific subtypes and variation in people with dementia or carers separately. To address limitations in the existing studies, the aim of this study is to investigate the potential impact of dementia subtypes on the capability to live well using 1283 dyads of community-dwelling people with dementia and their carers in Great Britain.

## Methods

### Study population

The Improving the experience of Dementia and Enhancing Active Life (IDEAL) project is a longitudinal cohort study of community-dwelling people with dementia and their carers across England, Scotland and Wales. The study was set up to investigate social, psychological and economic factors that support people living well with dementia. The participants were recruited through a network of 29 NHS sites between July 2014 and August 2016. Eligible participants needed to have a clinical diagnosis of dementia and a Mini-Mental State Examination (MMSE) score of 15 or above on entry into the study. Primary carers, who provided practical or emotional unpaid support for people with dementia, were also invited to take part where possible. For those who agreed to take part, researchers visited participants and completed structured interviews to collect data. The study protocol has been published elsewhere [14]. The IDEAL study was approved by the Wales Research Ethics Committee 5 (reference 13/WA/0405) and the Ethics Committee of the School of Psychology, Bangor University (reference 2014-11684). IDEAL is registered with the UK Clinical Research Network, number 16593.

The IDEAL cohort at baseline included 1547 people with dementia and 1283 carers. This analysis focused on the 1283 dyads of person with dementia and their carer.

### Measurements

Capability to live well was defined using three individual measures for quality of life, life satisfaction and wellbeing for the person with dementia and the carer. For people with dementia, self-rated life satisfaction was measured by the Satisfaction with Life Scale (SwLS; score range 5–35), which is designed to measure global judgements of satisfaction with life [15]; wellbeing was measured by the World Health Organization Five Well-being Index (WHO-5; score range 0–100), which includes items on positive mood, vitality and general interests [16]; and quality of life was measured by the Quality of Life in Alzheimer’s Disease (QOL-AD; score range 13–52), which is a dementia-specific measure of quality of life, incorporating multiple aspects of mood, health status, interpersonal relationships and financial situation [17]. The same measures for life satisfaction and wellbeing were used in the carer interview, but quality of life was measured using the World Health Organization Quality of Life-Brief (WHOQOL-BREF), which includes two single indicators (overall quality of life and general health) and four domains (physical health, psychological health, social relationships and environment) [18]. WHOQOL-BREF is designed to measure multiple components related to quality of life and does not have a total score. To provide an overall score for quality of life in carers, a factor analysis was conducted to estimate factor scores for those with complete data. The mean and standard deviation (SD) of the WHOQOL-BREF factor score was 0.0 (SD = 2.1) with a range between − 7.9 and 4.7. More detailed information is provided in Additional file 1.

The interviews collected information on age, sex, dementia subtypes and the type of relationship between the person with dementia and the carer. Age was divided into five groups: < 65, 65–69, 70–74, 75–79 and ≥ 80 for both people with dementia and carers. Dementia diagnoses were obtained from medical records of the participants and classified in seven groups: Alzheimer’s disease (AD), vascular dementia (VaD), mixed AD and VaD, frontotemporal dementia (FTD), Parkinson’s disease dementia (PDD), Lewy body dementia (LBD) and other/unspecified. For those who selected other or an unspecified diagnosis in the interviews, open-ended text descriptions provided by the interviewer were reviewed by two clinicians and re-categorised into the six empirical groups where possible. The type of relationship between the person with dementia and carer was categorised into two groups: spouse/partner and family/friend such as daughters, sons and grandchildren. Due to the small numbers of friends serving as carers (*N* = 12), this group was combined with family carers. As poor health status has been related to poor quality of life and wellbeing, the number of chronic conditions was used to indicate the general physical health of people with dementia and was measured using the Charlson Comorbidity Index [19].

### Analytical strategy

**Fig. 1:**
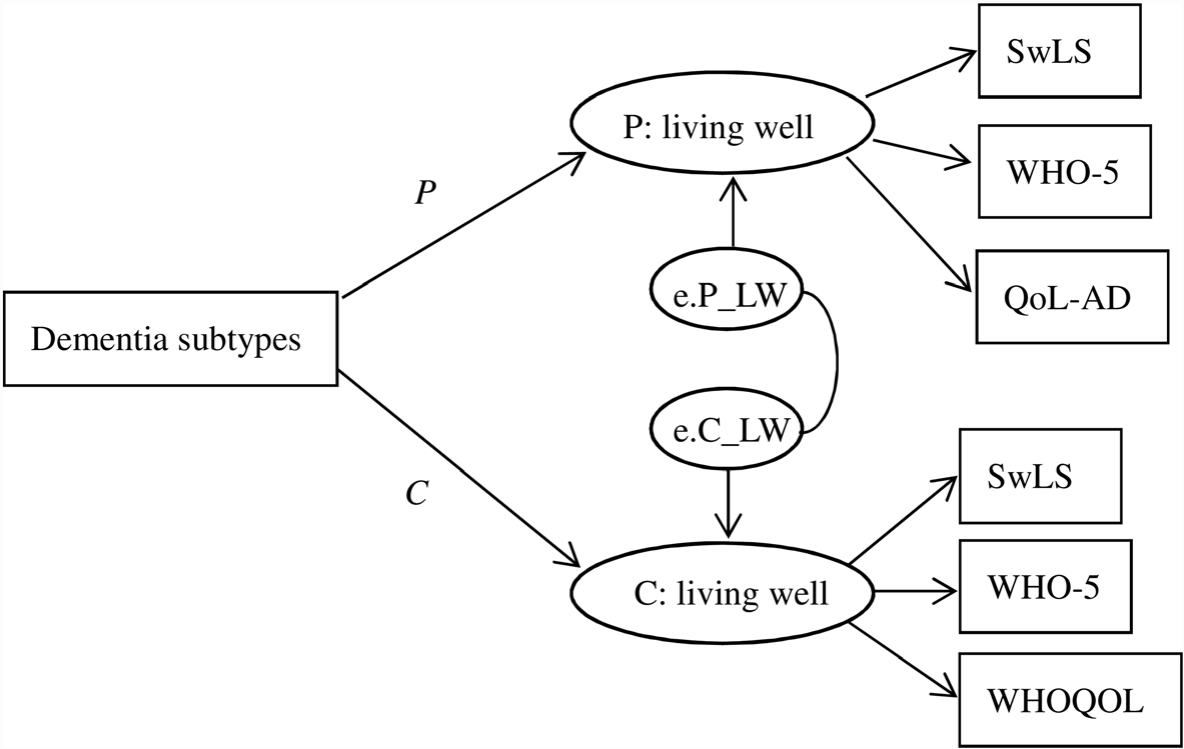
Dyadic relationship between subtypes and capability to live well in people with dementia (*P*) and carers (*C*)

Before conducting the dyadic analysis, the associations between subtypes and the three living well measures in people with dementia and carers were investigated using multivariate modelling. Structural equation modelling (SEM) was used to build two latent factors including three living well measures in people with dementia (SwLS, WHO-5 and QOL-AD) and carers (SwLS, WHO-5 and WHOQOL-BREF factor score), and SwLS was fixed at 1 in the latent factors. Covariance of their error terms was estimated to account for the dyadic structure (Fig. 1). The dyadic relationships between subtypes and living well latent factors (*P* and *C* in Fig. 1) were also examined in SEM adjusting for the age and sex of people with dementia and carers as well as the type of relationship between persons with dementia and their carers. Further adjustment for number of chronic conditions was conducted to account for physical health conditions in people with dementia. The estimation method of maximum likelihood with missing values was used in the modelling in order to account for missing data [20]. To investigate whether the associations between dementia subtypes and capability to live well were different in those without carers, a sensitivity analysis was conducted to test the model in all IDEAL participants (*N* = 1547). This study was based on the IDEAL baseline data version 2.0. All analyses were conducted using Stata 14.2.

## Results

Descriptive information on the 1283 dyads is reported in Table 1. The median age was 77 (interquartile range (IQR) = 12.0) for people with dementia and 71 (IQR = 14.0) for carers. More than half of people with dementia were men, while nearly 70% of the carers were women. Most carers were spouses or partners (81%) of the person with dementia. Nearly 56% of the participants had AD, 11% had VaD and 21% had a diagnosis of mixed AD and VaD. A relatively small percentage of participants had FTD (3.5%), PDD (3.4%) and LBD (3.4%); the other/unspecified category accounted for 2.5%. The median MMSE score of people with dementia was 23 (IQR = 6.0), and 27% of people with dementia and 22% of the carers had no educational qualification. Approximately one-third of people with dementia (35%) and carers (30%) reported fair or poor self-rated health.

**Table 1:** Descriptive information on the study population (*N* = 1283)

	People with dementia: *N*(%)	Carers: *N*(%)
Age		
80+	482 (37.6)	216 (16.8)
75–79	306 (23.9)	223 (17.4)
70–74	232 (18.1)	267 (20.8)
65–69	160 (12.5)	208 (16.2)
< 65	103 (8.0)	369 (28.8)
Sex		
Men	755 (58.9)	402 (31.3)
Women	528 (41.1)	881 (68.7)
Dementia subtype		
Alzheimer’s disease (AD)	715 (55.7)	
Vascular dementia (VaD)	142 (11.1)	
Mixed AD and VaD	263 (20.5)	
Frontotemporal dementia (FTD)	45 (3.5)	
Parkinson’s disease dementia (PDD)	43 (3.4)	
Lewy body dementia (LBD)	43 (3.4)	
Other/unspecified	32 (2.5)	
Type of relationship		
Spouse/partner	1039 (81.0)	
Family/friend	244 (19.0)	
Chronic conditions (missing = 89)		
1–2	611 (51.2)	
3–4	426 (35.7)	
5+	157 (13.1)	

Table 2 reports the means and standard deviations of the three living well measures in people with dementia and carers across subtypes. People with dementia generally reported higher scores for life satisfaction and wellbeing than their carers, but the patterns across subtypes were similar in people with dementia and carers. The mean scores were generally higher in AD and lower in PDD and LBD.

**Table 2:** Mean scores and standard deviation of living well measures in people with dementia and carers across subtypes

	People with dementia			Carers		
	SwLS	WHO-5	QOL-AD	SwLS	WHO-5	WHOQOL-BREF^a^
AD	27.3 (5.5)	64.2 (19.5)	37.7 (5.5)	24.1 (6.4)	56.6 (19.6)	0.1 (2.0)
VaD	25.6 (6.3)	58.6 (21.2)	35.9 (6.5)	23.4 (6.3)	53.0 (19.3)	−0.1 (2.1)
Mixed AD/VaD	26.3 (5.9)	59.8 (21.0)	36.3 (5.8)	24.4 (6.5)	55.2 (19.9)	0.0 (2.1)
FTD	25.7 (5.9)	61.0 (20.5)	38.7 (5.4)	21.9 (7.2)	53.2 (21.8)	−0.2 (2.2)
PDD	22.0 (6.8)	47.9 (20.4)	33.1 (5.7)	21.5 (5.6)	50.1 (19.0)	−0.4 (1.8)
LBD	23.7 (5.2)	50.7 (17.8)	33.0 (6.3)	20.4 (7.9)	47.7 (20.9)	−0.7 (2.1)
Other	26.2 (7.6)	58.5 (24.8)	34.7 (8.1)	23.2 (6.4)	56.9 (18.3)	−0.4 (2.2)

*AD* Alzheimer’s disease, *VaD* vascular dementia, *FTD* frontotemporal dementia, *PDD* Parkinson’s disease dementia, *LBD* Lewy body dementia, *SwLS* Satisfaction with Life Scale, *WHO-5* World Health Organization Five Well-being Index, *QOL-AD* Quality of Life in Alzheimer’s Disease,

^a^WHOQOL-BREF factor score estimated from six domains

In people with dementia, loadings of the living well latent factors (SwLS fixed at 1) were estimated to be 3.82 (95% confidence interval (CI) 3.52, 4.13) for WHO-5 and 1.21 (95% CI 1.12, 1.31) for QOL-AD. In carers, loading estimates were 3.37 (95% CI 3.11, 3.62) for WHO-5 and 0.41 (95% CI 0.38, 0.44) for the WHOQOL-BREF factor score. The two living well latent factors were correlated, and the estimated covariance was 5.62 (95% CI 4.22, 7.03).

Table 3 reports dyadic associations between subtypes and capability to live well in people with dementia and carers (P and C in Fig. 1). Participants with non-AD subtypes reported a lower capability to live well than those with AD. Significant differences were found for VaD (− 1.69; 95% CI – 2.52, − 0.87), mixed AD/VaD (− 1.34; 95% CI – 1.99, − 0.70), PDD (− 4.39; 95% CI – 5.80, − 2.97), LBD (− 3.81; 95% CI – 5.23, − 2.40) and other (− 1.98; 95% CI – 3.59, − 0.38) after adjusting for age, sex and type of relationship. For carers, the variations across subtypes were relatively small, but lower capability to live well was also found for carers of people with PDD (− 1.55; 95% CI – 3.06, − 0.03) and LBD (− 1.77; 95% CI – 3.29, − 0.25) compared to carers of people with AD. Further adjustment for the number of chronic conditions in people with dementia attenuated the difference in VaD (− 0.96; 95% CI – 1.77, − 0.15) and mixed AD/VaD (− 0.87; 95% CI – 1.50, − 0.24) for people with dementia, but the effect sizes for PDD and LBD remained similar in both. The association between subtypes and capability to live well was similar for all people with dementia, including those without carers. More detailed information on the sensitivity analysis is provided in Additional file 1.

**Table 3:** Results of structural equation model: dyadic associations between subtypes and capability to live well in people with dementia and carers (*N* = 1283)

	Unadjusted		Adjusted 1		Adjusted 2	
	Person with dementia (*P*)	Carer (*C*)	Person with dementia (*P*)	Carer (*C*)	Person with dementia (*P*)	Carer (*C*)
AD	(Reference)	(Reference)	(Reference)	(Reference)	(Reference)	(Reference)
VaD	−1.64 (−2.47, −0.82)	−0.72 (−1.62, 0.17)	− 1.69 (− 2.52, − 0.87)	−0.58 (− 1.47, 0.31)	−0.96 (− 1.77, − 0.15)	−0.22 (− 1.12, 0.68)
Mixed AD/VaD	− 1.14 (− 1.78, − 0.50)	−0.17 (− 0.87, 0.54)	−1.34 (− 1.99, − 0.70)	−0.19 (− 0.89, 0.50)	−0.87 (− 1.50, − 0.24)	0.05 (− 0.66, 0.75)
FTD	−0.13 (− 1.49, 1.23)	−0.83 (− 2.34, 0.68)	0.39 (− 0.99, 1.78)	−0.30 (− 1.80, 1.20)	0.17 (−1.16, 1.50)	−0.42 (− 1.92, 1.07)
PDD	−4.26 (−5.68, − 2.85)	−1.71 (− 3.24, − 0.18)	−4.39 (− 5.80, − 2.97)	− 1.55 (− 3.06, − 0.03)	− 4.28 (− 5.65, − 2.91)	− 1.51 (− 3.02, − 0.01)
LBD	−3.72 (− 5.14, − 2.31)	−2.29 (− 3.84, − 0.75)	− 3.81 (− 5.23, − 2.40)	− 1.77 (− 3.29, − 0.25)	−3.76 (− 5.14, − 2.39)	− 1.77 (− 3.28, − 0.26)
Other	− 1.88 (− 3.48, − 0.27)	− 0.84 (− 2.61, 0.93)	−1.98 (− 3.59, − 0.38)	− 0.97 (− 2.72, 0.78)	−1.98 (− 3.54, − 0.43)	−0.99 (− 2.72, 0.75)

*AD* Alzheimer’s disease, *VaD* vascular dementia, *FTD* frontotemporal dementia, *PDD* Parkinson’s disease dementia, *LBD* Lewy body dementia, *Adjusted 1* age and sex in people with dementia and carers, type of relationship between the person with dementia and carer, *Adjusted 2* all factors in Adjusted 1 and number of chronic conditions in people with dementia

**Fig. 2:**
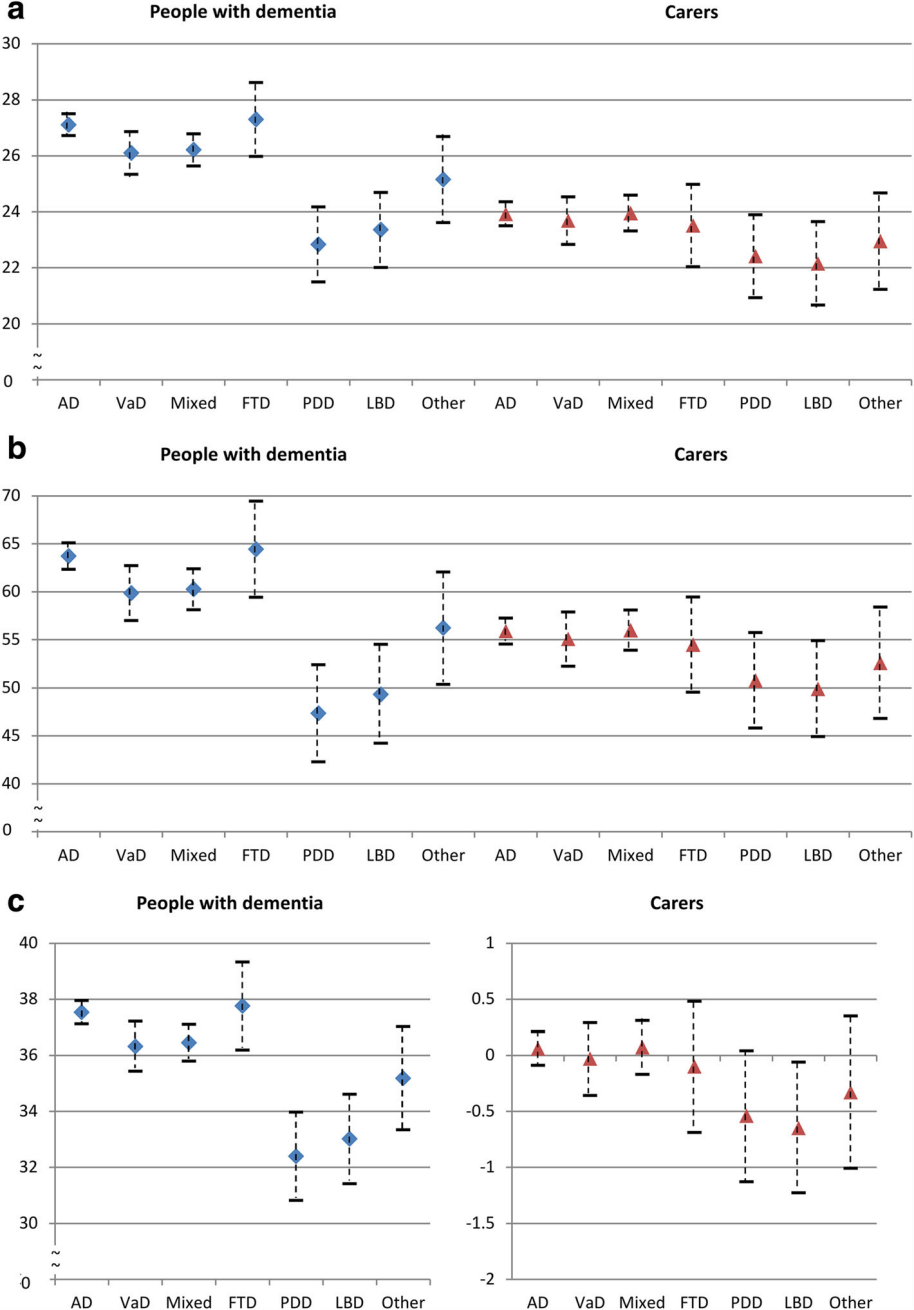
Estimated scores of living well measures across subtypes (adjusted for age, sex, type of relationship and number of chronic conditions in people with dementia) (**a**) Life satisfaction (SwLS) (**b**) Well-being (WHO-5) (**c**) Quality of life (QOL-AD) for people with dementia, WHOQOL-BREF factor score for carers

Based on the adjusted results, Fig. 2a–c shows estimated scores for the three living well measures across subtypes in people with dementia and their carers. Carers had systematically lower scores than people with dementia, but estimates for SwLS and WHO-5 were similar in those with PDD and LBD.

## Discussion

### Main findings

Using dyadic analysis methods, this study suggest a potential impact of subtype diagnosis on capability to live well in both people with dementia and carers. People with non-AD subtypes, including VaD, mixed VaD/AD, PDD and LBD, had a lower capability to live well than those with AD. For carers, those caring for people with PDD and LBD reported lower scores on living well measures than carers of people with AD. Further adjustment for comorbidity attenuated differences between AD, VaD and mixed AD/VaD, but PDD and LBD continued to have a particularly strong impact on capability to live well in both people with dementia and carers.

### Strengths and limitations

The IDEAL study included a large number of community-dwelling people with dementia and their carers across Great Britain. In addition to major subtypes (AD and VaD), people with rare subtypes were also recruited and were represented by at least 40 dyads in this study population. The interviews included multiple measures of living well, including aspects of quality of life, life satisfaction and wellbeing for both people with dementia and their carers. The method of dyadic analysis was used to investigate the association between subtypes and capability to live well in both people with dementia and carers and to take into account correlations within dyads.

To be eligible to take part, participants needed to have a clinical diagnosis of dementia and a MMSE score of 15 or above. People with severe dementia were not included in the study, and the dyadic association between subtypes and capability to live well might be different in this group compared to the current study population focusing on mild to moderate dementia. Given our interest in the dyadic relationship, this analysis mainly focused on participants with carers. Since those without carers might have better health status and functional ability and in some cases might not need a carer, the association between subtypes and capability to live well might be different in this group. Nevertheless, similar results were found in sensitivity analyses including all participants irrespective of carer involvement (*N* = 1547); therefore, this should have a minimal impact on the main findings.

The diagnoses of dementia subtypes were made by different clinicians across the country. Variation in clinical practice and potential diagnostic misclassification could not be addressed in this analysis. However, a clinical diagnosis reflects the experience of people with dementia and their carers attending health services and leads to selection of particular treatments and disease management plans. Different measures for quality of life were used in people with dementia and the carers, and therefore changes in quality of life scores were not directly comparable. To generate an overall score for quality of life in carers, six independent domains in WHOQOL-BREF were combined using factor analysis. Although measures for quality of life were different for people with dementia and carers, this study mainly focused on relative differences, and similar patterns were found in these two quality of life measures.

### Interpretation of results

People with non-AD subtypes had a lower capability to live well than those with AD. When further adjusting for comorbidity, the difference between AD, VaD and mixed AD/VaD reduced considerably. This suggests that the burden of chronic conditions might explain much of the observed differences across these subtypes. Although the diagnosis of VaD is based on cerebrovascular pathology and small vessel disease, people with VaD might also experience physical disabilities as well as language and visuospatial deficits due to stroke and heart attack and thus may have poor quality of life and wellbeing [8].

People with PDD and LBD continued to show a particularly poor capability to live well, even when we further adjusted for the number of chronic conditions. This corresponds to findings from previous studies [9–11]. These two subtypes are closely related, and the differential diagnosis is largely based on when symptoms first appear. The core symptoms of LBD, including fluctuating cognition, visual hallucinations and spontaneous features of Parkinsonism [8], may have a relatively large impact on daily life compared to the major symptoms of memory loss in AD. A higher number of autonomic symptoms such as fatigue, postural dizziness and mucosal dryness have also been reported in PDD and LBD than in AD [21]. PDD is also associated with high comorbidity [22, 23], and the mix of physical, emotional and cognitive changes might have a negative impact on quality of life for the person with dementia as well as their carer. Carers of people with PDD and LBD have been reported to experience a higher level of stress than carers of those with AD and VaD [24]. This might be due to the challenges of responding to symptoms such as hallucinations, motor disability and functional impairment in PDD and LBD [11].

The literature has suggested a greater burden of care in FTD [12, 13]. FTD generally occurs in younger age groups (< 65) and includes symptoms related to changes in personality, behaviour and function [7]. Although these symptoms may plausibly increase the burden of caregiving and cause poor quality of life and wellbeing, this study did not find a difference in the capability to live well between carers of people with FTD and AD. A previous study from Australia has reported variation in caregiving burden across different FTD variants [12]. Although carers of persons with behavioural variant FTD (bvFTD) did report a particularly high burden, carers for people with other FTD variants, including semantic dementia and progressive non-fluent aphasia, had similar levels of burden to carers of people with AD. Indeed, the mean scores for living well measures were found to be lower for the 10 IDEAL carers of people with bvFTD than for carers of people with other FTD variants. However, it is not possible to test differences within this subtype based on such a small sample size and limited statistical power. In addition, this study population only included those with mild to moderate dementia and did not include those with advanced FTD.

Dyadic modelling was used to consider the capability to live well in both people with dementia and carers and to account for correlations within dyads. In addition to relative differences across subtype, this study also reveals baseline differences between people with dementia and carers through this dyadic analysis approach. Capability to live well was clearly not independent in these dyads, but relative difference across subtype, a dyad-level measure, was not considerably affected by this correlation. However, absolute scores on living well measures were lower for carers than for people with dementia. Despite variation across subtypes, baseline scores for life satisfaction and wellbeing in carers were close to those for participants with PDD and LBD, who had the worst capability to live well. This indicates that carers generally reported a poorer capability to live well than people with dementia, regardless of subtype.

### Clinical implications and future research directions

Variation in capability to live well was found across dementia subtypes, particularly PDD and LBD. Guidelines for dementia care may need to be tailored for different subtypes and provide additional support for these high-risk groups. Since the impact of living with VaD and mixed AD/VaD may be related to comorbidity, treatment of hypertension and vascular diseases is important for those with these subtypes. Health professionals who provide post-diagnostic support and care may need to pay more attention to the PDD and LBD subtypes and consider potential approaches to improve quality of life and wellbeing for both people with dementia and their carers [25]. In addition to medical treatments, some non-pharmacological interventions might support those living with PDD and LBD to maintain daily function. For example, a recent pilot randomised controlled trial focusing on 29 people with PDD and LBD has suggested that cognitive rehabilitation, an individualised approach addressing personally relevant goals, can be effective in managing the impact of the cognitive difficulties on daily life and in improving quality of life [26]. Future intervention studies may extend this approach to include the physical symptoms seen in PDD and LBD and reduce the combined impact of cognitive and physical symptoms on the capability to live well with these conditions. Carers reported relatively low scores on living well measures across all dementia subtypes. Burden of caregiving appears to be an important issue, and appropriate support for family carers is vital [1].

To reduce the impact of subtype diagnosis, future research may focus on identifying specific factors related to the quality of life and wellbeing in PDD and LBD and developing potential interventions to improve disease management in people with dementia and carers. In addition to medications, psychosocial and rehabilitative interventions may play an important role in addressing neuropsychiatric and behavioural symptoms and general physical, psychological and social health [27].

## Conclusions

The findings of this study suggest important differences in the capability to live well across dementia subtypes. Dementia care and health professionals who provide post-diagnostic support should consider different needs across subtypes, in particular the complex needs of people living with PDD and LBD and their carers.

## Additional file

Additional file 1: **Table S1.1.** Mean and standard deviation for WHOQOL-BREF by carer age, sex, dementia subtypes and type of relationship between person with dementia and carer (*N* = 1283). **Table S1.2.** Loadings of six WHOQOL-BREF domains. **Figure S1.** Histogram of WHOQOL-BREF factor score. **Table S2.1.** Multivariate modelling of living well measures and subtypes in all people with dementia (*N* = 1363; adjusted for age, sex and type of relationship). **Table S2.2.** The association between living well and subtypes in people with dementia. (PDF 42 kb)

## Abbreviations

AD: Alzheimer’s disease
bvFTD: Behavioural variant frontotemporal dementia
CI: Confidence interval
FTD: Frontotemporal dementia
IDEAL: Improving the experience of Dementia and Enhancing Active Life
IQR: Interquartile range
LBD: Lewy body dementia
MMSE: Mini-Mental State Examination
PDD: Parkinson’s disease dementia
QOL-AD: Quality of Life in Alzheimer’s Disease
SD: Standard deviation
SEM: Structural equation modelling
SwLS: Satisfaction with Life Scale
VaD: Vascular dementia
WHO-5: World Health Organization Five Well-being Index
WHOQOL-BREF: World Health Organization Quality of Life-Brief
