# Supplementary material for: Dementia subtype and living well: results from the Improving the experience of Dementia and Enhancing Active Life (IDEAL) study
Source: BMC Med. 2018 Sep 11;16:140. doi: 10.1186/s12916-018-1135-2 (PMC6131832; doi:10.1186/s12916-018-1135-2)
Supplement: Supplementary file 1 — Table S1.1. Mean and standard deviation for WHOQOL-BREF by carer age, sex, dementia subtypes and type of relationship between person with dementia and carer (N = 1283). Table S1.2. Loadings of six WHOQOL-BREF domains. Figure S1. Histogram of WHOQOL-BREF factor score. Table S2.1. Multivariate modelling of living well measures and subtypes in all people with dementia (N = 1363; adjusted for age, sex and type of relationship). Table S2.2. The association between living well and subtypes in people with dementia. (PDF 42 kb) [file 12916_2018_1135_MOESM1_ESM.pdf]

Dementia subtype and living well: results from the Improving the experience of Dementia and Enhancing Active Life (IDEAL) study

Additional files

1. Factor score for WHOQOL-BREF

2. Sensitivity analysis: models for all people with dementia

1. Factor score for WHOQOL-BREF

Table S1.1 shows distributions of six WHOQOL-BREF domain scores by carer demographic factors and dementia subtypes. The six domain scores were fitted in one structural equation model and factor scores were estimated for those with complete data (N=1233). The loadings of six domains are reported in Table S1.2. The mean and standard deviation of the factor score was 0.0 (2.1) with a range between -7.9 and 4.7 (Figure S1).

Table S1.1: Mean and standard deviation for WHOQOL-BREF by carer age, sex, dementia subtypes and type of relationship between person with dementia and carer (N=1283)

	N (%)	Overall QOL	General health	Physical	Psychological	Social relationships	Environment
Age							
<65	369 (29)	3.8 (0.9)	3.4 (1.1)	15.6 (3.2)	14.5 (2.6)	14.7 (3.1)	15.8 (2.4)
65-69	208 (16)	3.9 (0.8)	3.4 (1.0)	15.5 (3.1)	15.1 (2.3)	14.6 (2.8)	16.5 (1.9)
70-74	267 (21)	3.7 (0.7)	3.4 (0.9)	15.1 (3.0)	15.0 (2.2)	14.7 (2.6)	16.3 (1.9)
75-79	223 (17)	3.9 (0.8)	3.5 (1.0)	15.0 (2.8)	15.2 (1.9)	15.1 (2.3)	16.3 (1.8)
80+	216 (17)	3.8 (0.8)	3.4 (0.9)	14.6 (2.8)	15.1 (2.1)	15.1 (2.5)	16.1 (2.0)
Sex							
Men	402 (31)	3.9 (0.8)	3.5 (1.0)	15.6 (2.9)	15.6 (2.2)	15.0 (2.8)	16.5 (2.0)
Women	881 (69)	3.8 (0.8)	3.4 (1.0)	15.0 (3.0)	14.6 (2.3)	14.8 (2.7)	16.0 (2.1)
Subtypes							
AD	715 (56)	3.9 (0.8)	3.4 (1.0)	15.3 (3.0)	15.0 (2.3)	14.8 (2.7)	16.3 (2.0)
VaD	142 (11)	3.8 (0.8)	3.4 (1.0)	15.1 (3.1)	14.8 (2.2)	14.7 (2.7)	15.9 (2.0)
Mixed	263 (20)	3.8 (0.8)	3.5 (1.0)	15.3 (3.1)	14.9 (2.4)	15.1 (2.8)	16.1 (2.1)
FTD	45 (4)	3.7 (0.8)	3.4 (1.0)	15.6 (3.1)	14.9 (2.3)	14.0 (3.0)	16.1 (2.1)
PDD	43 (3)	3.6 (0.7)	3.3 (0.9)	14.7 (2.4)	14.6 (2.1)	14.8 (1.9)	15.7 (1.9)
LBD	43 (3)	3.4 (0.9)	3.3 (1.1)	14.7 (2.9)	14.1 (2.3)	14.4 (2.7)	15.7 (1.8)
Other	32 (3)	3.7 (0.7)	3.3 (1.2)	14.5 (3.2)	14.9 (2.3)	14.1 (3.0)	15.7 (2.6)
Relationships							
Spouse/partner	1039 (81)	3.8 (0.8)	3.4 (1.0)	15.0 (3.0)	14.9 (2.2)	14.7 (2.6)	16.2 (2.0)
Family/friend	244 (19)	4.0 (0.9)	3.6 (1.1)	16.0 (3.0)	14.9 (2.6)	15.3 (3.2)	16.1 (2.4)

Table S1.2: Loadings of six WHOQOL-BREF domains

	Loadings
Physical health	1 (fixed)
Psychological health	0.84 (0.77, 0.91)
Social relationship	0.75 (0.67, 0.83)
Environment	0.67 (0.61, 0.73)
General health	0.25 (0.23, 0.28)
Overall quality of life	0.31 (0.28, 0.33)

Figure S1: Histogram of WHOQOL-BREF factor score

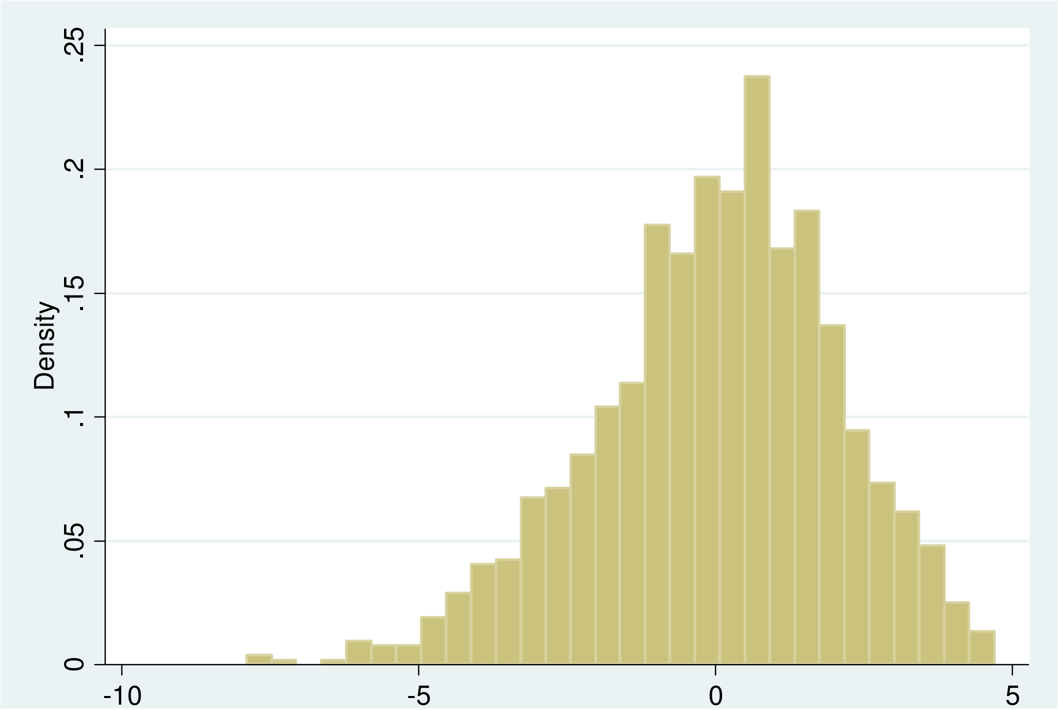

2. Sensitivity analysis: models for all people with dementia

Table S2.1 reports results of multivariate modelling based on all 1547 people with dementia. Compared to AD, lower scores for three living well outcomes were found in VaD, mixed AD/VaD, PDD and LBD. Participants without carers also reported lower living well scores than those with spouse/partner carers. Table S2.2 reports the SEM results using estimation method of maximum likelihood with missing value (mlmv). The unadjusted and adjusted results were similar in all people with dementia (N=1547) and those with carers (N=1283) as well as estimates from dyadic modelling.

Table S2.1: Multivariate modelling of living well measures and subtypes in all people with dementia (N=1363; adjusted for age, sex and type of relationship)

	SwLS	WHO-5	QoL-AD
Subtypes			
AD (ref.)	-	-	-
VaD	-1.83 (-2.84, -0.81)	-7.45 (-10.99, -3.92)	-2.39 (-3.42, -1.37)
Mixed AD/VaD	-1.13 (-1.90, -0.35)	-5.16 (-7.87, -2.45)	-1.55 (-2.34, -0.77)
FTD	-0.48 (-2.20, 1.23)	0.72 (-5.23, 6.67)	1.72 (-0.01, 3.44)
PDD	-5.53 (-7.38, -3.68)	-16.06 (-22.49, -9.63)	-4.96 (-6.82, -3.10)
LBD	-3.82 (-5.56, -2.08)	-14.00 (-20.06, -7.93)	-4.71 (-6.47, -2.95)
Other	-0.46 (-2.48, 1.56)	-1.37 (-8.40, 5.65)	-2.56 (-4.60, -0.53)
Age			
≥80 (ref.)	-	-	-
75-79	-0.38 (-1.18, 0.43)	-2.42 (-5.21, 0.37)	-0.16 (-0.97, 0.65)
70-74	-1.47 (-2.38, -0.56)	-3.79 (-6.95, -0.62)	-0.64 (-1.56, 0.27)
65-69	-1.58 (-2.62, -0.55)	-5.96 (-9.57, -2.36)	-1.24 (-2.28, -0.19)
<65	-4.84 (-6.00, -3.68)	-9.56 (-13.58, -5.53)	-3.09 (-4.26, -1.93)
Sex			
Men (ref.)	-	-	-
Women	-0.01 (-0.66, 0.63)	-1.90 (-4.14, 0.34)	0.21 (-0.41, 0.89)
Type of relationship			
Spouse/partner (ref.)	-	-	-
Family/friend	-1.89 (-2.81, -0.98)	-3.29 (-6.46, -0.11)	-1.39 (-2.31, -0.47)
No carers	-2.72 (-3.57, -1.88)	-2.68 (-5.63, 0.26)	-0.87 (-1.73, -0.02)

Table S2.2: The association between living well and subtypes in people with dementia

	All people with dementia (N=1547)		Dyads of people with dementia and carers (N=1283)	
	Adjusted 1	Adjusted 2	Adjusted 1	Adjusted 2
Measurement model				
SwLS	1 (fixed)	1 (fixed)	1 (fixed)	1 (fixed)
WHO-5	3.72 (3.45, 3.99)	3.75 (3.48, 4.02)	3.83 (3.53, 4.14)	3.87 (3.56, 4.18)
QoL-AD	1.13 (1.04, 1.21)	1.14 (1.06, 1.23)	1.18 (1.08, 1.27)	1.19 (1.09, 1.28)
Structural model				
AD (ref.)	-	-	-	-
VaD	-2.26 (-3.04, -1.48)	-1.43 (-2.20, -0.67)	-1.69 (-2.52, -0.86)	-0.96 (-1.77, -0.15)
Mixed AD/VaD	-1.38 (-1.98, -0.78)	-0.85 (-1.44, -0.27)	-1.36 (-2.01, -0.71)	-0.89 (-1.52, -0.26)
FTD	0.77 (-0.54, 2.07)	0.55 (-0.71, 1.80)	0.34 (-1.05, 1.73)	0.12 (-1.22, 1.46)
PDD	-4.58 (-6.01, -3.14)	-4.44 (-5.83, -3.06)	-4.45 (-5.86, -3.03)	-4.33 (-5.70, -2.96)
LBD	-4.09 (-5.41, -2.77)	-4.07 (-5.35, -2.80)	-3.79 (-5.21, -2.36)	-3.74 (-5.11, -2.37)
Other	-1.79 (-3.26, -0.33)	-1.82 (-3.23, -0.40)	-2.00 (-3.61, -0.39)	-1.98 (-3.54, -0.43)

Adjusted 1: adjusted for age, sex and type of relationship; Adjusted 2: adjusted for adjusted for age, sex, type of carer relationships and number of chronic conditions in people with dementia
